# Factors influencing Meropenem utilization as the drug of choice in patients with pneumonia at a referral hospital in Makassar

**DOI:** 10.1017/ash.2025.149

**Published:** 2025-09-03

**Authors:** Jessica Kwenandar, Sudirman Katu, Risna Halim Mubin, Ariantin Ulfah Said Culla

**Affiliations:** Department of Tropical Medicine and Infectious Disease, Faculty of MedicineHasanuddin University, Makassar, Indonesia

**Keywords:** Meropenem, antibiotic utilization, pneumonia

## Abstract

**Objectives:** Meropenem has become one of the most widely used antibiotics and is considered to be the drug of choice for empirical treatment in patients with pneumonia. The aim of this study is to evaluate factors associated with the use of Meropenem as a broad-spectrum antibiotic in a referral hospital in Makassar. **Methods:** In a retrospective observational study we conducted over one-month period (January- February 2024), adult patients diagnosed with pneumonia who received Meropenem were selected. We included data such as length of stay, admission to the intensive care unit, use of ventilator, basis of prescription (either empirical or culture-based), and laboratory profiles such as white blood cell count, procalcitonin levels, blood culture and resistance towards antibiotics. **Results:** Over one-month period, thirty patients admitted to our hospital with pneumonia were evaluated. Among these patients, several factors such as admission in intensive care unit, use of ventilator, and procalcitonin levels showed statically significance (p < 0,05) while blood culture and antibiotic resistance showed minimal impact towards utilization of Meropenem in patients with pneumonia. **Conclusions:** In conclusion, our study indicates that Meropenem usage for pneumonia treatment is significantly influenced by admission to the intensive care unit, use of ventilator, and specific laboratory parameters such as procalcitonin levels. Further research with larger scale is needed to evaluate utilization of Meropenem in clinical practices.

